# The Upper Extremity Flexion Synergy Is Minimally Expressed in Young Individuals With Unilateral Cerebral Palsy Following an Early Brain Injury

**DOI:** 10.3389/fnhum.2020.590198

**Published:** 2020-10-16

**Authors:** Nayo M. Hill, Julius P. A. Dewald

**Affiliations:** ^1^Department of Biomedical Engineering, Northwestern University, Evanston, IL, United States; ^2^Department of Physical Therapy and Human Movement Sciences, Northwestern University, Chicago, IL, United States; ^3^Department of Physical Medicine and Rehabilitation, Northwestern University, Chicago, IL, United States

**Keywords:** cerebral palsy, perinatal stroke, reaching, flexion synergy, pediatric hemiparesis, independent joint control

## Abstract

Hemiparetic stroke in adulthood often results in the grouped movement pattern of the upper extremity flexion synergy thought to arise from an increased reliance on cortico-reticulospinal pathways due to a loss of lateral corticospinal projections. It is well established that the flexion synergy induces reaching constraints in individuals with adult-onset hemiplegia. The expression of the flexion synergy in individuals with brain injuries onset earlier in the lifespan is currently unknown. An early unilateral brain injury occurring prior to six months post full-term may preserve corticospinal projections which can be used for independent joint control and thus minimizing the expression of the flexion synergy. This study uses kinematics of a ballistic reaching task to evaluate the expression of the flexion synergy in individuals with pediatric hemiplegia (PH) ages six to seventeen years. Fifteen individuals with brain injuries before birth (*n* = 8) and around full-term (*n* = 7) and nine age-matched controls with no known neurological impairment completed a set of reaches in an admittance controlled robotic device. Descending drive, and the possible expression of the upper extremity flexion synergy, was modulated by increasing shoulder abduction loading. Individuals with early-onset PH achieved lower peak velocities when reaching with the paretic arm compared to controls; however, no differences in reaching distance were found between groups. Relative maintenance in reaching seen in individuals with early brain injuries highlights minimal expression of the flexion synergy. We interpret this conservation of independent control of the paretic shoulder and elbow as the use of more direct corticospinal projections instead of indirect cortico-reticulospinal pathways used in individuals with adult-onset hemiplegia.

## Introduction

Hemiplegia results from a unilateral lesion to the developing (pediatric-onset) or mature (adult-onset) brain and can lead to impairments to upper limb function in the impaired or paretic arm. A perinatal stroke acquired in utero occurring in one in 4000 live births (Lynch and Nelson, 2001; Hunt and Inder, 2006), is a main contributing cause of pediatric hemiplegia (PH) and often leads to a diagnosis of hemiparetic cerebral palsy (Novak, 2014). Cerebral palsy by definition describes a group of disorders from injury to the fetal or infant brain (Rosenbaum et al., 2007) and can present with weakness (Sanger et al., 2006; Mockford and Caulton, 2010; Sukal-Moulton et al., 2014a) and spasticity (Koman et al., 2013; Russo et al., 2019). In adulthood, ischemic or hemorrhagic stroke is a leading cause of long-term upper extremity disability (Broeks et al., 1999). Impairment is believed to be especially caused by the expression of the “flexion synergy” described by Brunnstrom (1970) as abnormal coupling of shoulder abduction (SABD) with shoulder extension and elbow, wrist, and finger flexion.

A hypothesized neural mechanism contributing to the expression of the flexion synergy is an increased reliance on indirect cortico-reticulospinal tracts in the absence of sufficient corticospinal resources (Cahill-Rowley and Rose, 2014; McPherson et al., 2018). Investigation of the flexion synergy in isometric conditions in individuals with adult-onset hemiplegia has revealed abnormal coupling between SABD and elbow, wrist, and finger flexion when measuring torques and muscle activity (Dewald et al., 1995, 2001; Beer et al., 1999; Dewald and Beer, 2001; McPherson and Dewald, 2019). In the same population, increased drive to shoulder abductors results in decreases in reaching area (Sukal et al., 2007), reaching distance (McPherson et al., 2018), and hand opening (Lan et al., 2017) in dynamic, multi-degree-of-freedom tasks. The current study explores whether this expression also exists in children and adolescents with PH given that corticospinal pathways are still maturing at the time of lesion (Eyre et al., 2001; Staudt, 2010).

In typical neurodevelopment, the initial bilateral corticospinal tract (CST) is pruned through a process of competitive inhibition. Ipsilateral projections are withdrawn and contralateral projections are strengthened as shown in the feline model (Martin, 2005) and human neonates and infants (Eyre et al., 2001). In humans, this pruning process takes place between 3 and 18 months of age (Eyre et al., 2001), and correlates with increasing motor skill. After a perinatal stroke, this typical pruning process can be interrupted, enabling the non-lesioned hemisphere to maintain more direct CST projections to motor neuron pools innervating both arms (Staudt, 2010). Thus, this may provide an alternative mechanism of neural control that does not result in an expression of the flexion synergy. Previous work in adolescents with PH during the generation of single-degree-of-freedom isometric maximum torques demonstrated a stratification in involuntary joint coupling patterns based on timing of lesion (Sukal-Moulton et al., 2014b). While there were some secondary joint torques produced in individuals with early-onset PH during a single-degree-of-freedom isometric task, it is currently unknown whether these individuals are able to move away from spontaneous joint torque coupling patterns during a functional, dynamic multi-degree-of-freedom task.

A ballistic forward reach with load on the shoulder abductors requires generation of torques outside of the flexion synergy pattern by combining SABD with horizontal shoulder flexion and elbow extension. SABD loading is an independent variable that appears to drive the expression of the flexion synergy in the paretic upper extremity (Sukal et al., 2007; McPherson et al., 2018). As a result, individuals with movement highly influenced by the flexion synergy are expected to have decreased success in completing this functional multi-degree-of-freedom task. The purpose of this study was to investigate whether individuals with PH who have sustained an early lesion to the developing brain express the upper extremity flexion synergy during ballistic reaching with SABD load modulation. In contrast to previous findings in adult-onset hemiplegia, we hypothesize that children and adolescents with early lesions, who may have a relative preservation of direct corticospinal projections either contralaterally or ipsilaterally, will have a reduced expression of the flexion synergy.

## Materials and Methods

### Participants

As demonstrated in previous work looking at timing (Sukal-Moulton et al., 2014a) or type of lesion (Kuczynski et al., 2018a, b), the stage of neural development at the time of lesion may result in differences in motor performance. Therefore, participants with PH were sorted into two groups of early lesions. Pre-natal (PRE) was defined as injury timing between the late second and early third trimesters of gestation and peri-natal (PERI) was defined as injury timing between the late third trimester until six months following full-term (Eyre et al., 2001). Individuals with PH were identified through the Cerebral Palsy Research Registry (Hurley et al., 2011), local clinics, and parent support groups. Inclusion criteria: (1) at least 6 years of age at time of testing, (2) unilateral motor impairment of the upper extremity resulting from a childhood-onset brain injury, and (3) ability to determine timing of injury by parent report, medical records, and/or brain imaging. Exclusion criteria: (1) reported use of medications suppressing the central nervous system such as baclofen; (2) botulinum toxin injections to any muscles in the upper extremity within 6 months of testing; (3) surgery on the paretic upper limb within 12 months of testing; or (4) cognitive dysfunction impairing ability to follow directions. A cohort of age- and gender-matched individuals with no history of neurological impairment [typical development (TD)] was recruited for comparison.

All participants or a parent provided informed and written consent to participate in this study which was approved by the Institutional Review Board of Northwestern University. The majority of those consented were minors who provided assent to participate.

### Clinical Assessments

To assess hand grip strength symmetry, a grip strength ratio between hands was calculated where a value of one indicates symmetry between hands. For the TD group, the ratio compared the non-dominant to the dominant hand. For the PH groups, the ratio compared the paretic (affected) to the non-paretic (less affected) hand. Grip strength was assessed in sitting by recording at least three maximal grip trials with the shoulder at 0° of abduction and elbow at 90° of flexion (Jamar Hand Dynamometer, B&L Engineering, Tustin, CA, United States). In addition to grip strength ratios, the cohort of individuals with PH were evaluated with a number of clinical assessments for descriptive analysis and functional comparison between injury timing groups. Function was classified using the Gross Motor Function Classification Scale (GMFCS) (Palisano et al., 1997; Rosenbaum et al., 2008) and the Manual Abilities Classification Scale (MACS) (Eliasson et al., 2006). Selective control of the arm and hand were evaluated using the Test of Arm Selective Control (TASC) (Krosschell et al., 2015; Sukal-Moulton et al., 2018) and the Fugl-Meyer Assessment-Upper Extremity (FMA-UE) (Fugl-Meyer et al., 1975; Fasoli et al., 2009). TASC score is a recently validated assessment implemented due to the appropriateness in evaluating the specific population being tested; it was not assessed for three participants (PRE, *n* = 3) because had not yet been validated at the time of testing. Information on patient perceived difficulty of unimanual and bimanual daily activities was acquired using the ABILHAND-Kids (under 18 years) or ABILHAND (over 18 years) (Penta et al., 2001; Arnould et al., 2004).

### Experimental Setup and Protocol

Both arms were tested for comparison and the first arm tested was varied in order to account for learning effects. In the TD group, the arms were defined as dominant or non-dominant based on writing hand. For the PH groups, arms were defined as non-paretic or paretic with the non-paretic hand corresponding to the writing hand.

**FIGURE 1:**
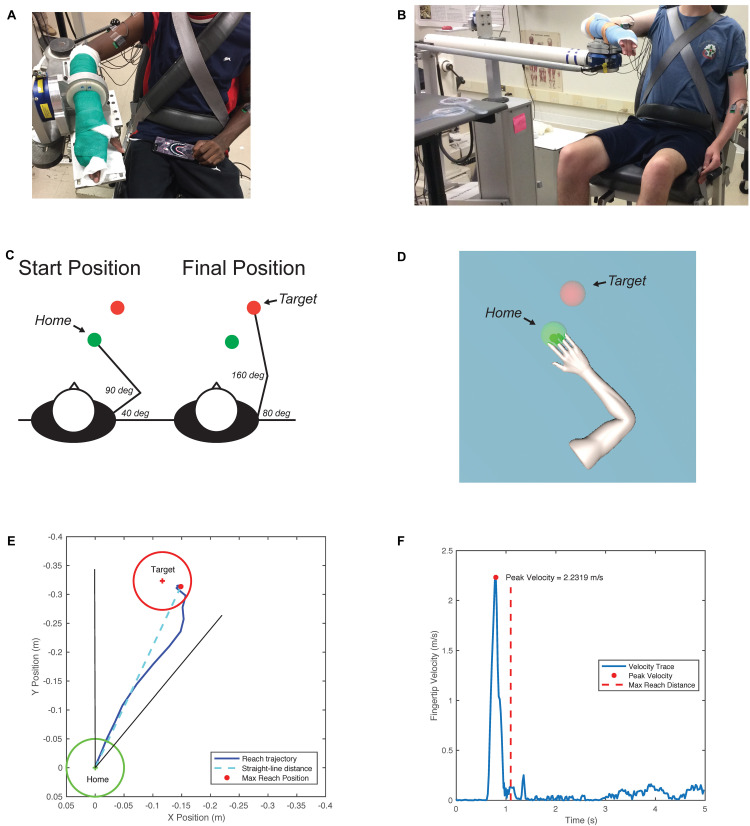
**(A)** Isometric setup for torque maxes. Participant with forearm attached to six-degree-of-freedom (DOF) load cell recording forces and torques along the *X*, *Y*, and *Z* axes. **(B)** ACT-3D setup for reaching tasks. Admittance controlled robot allows movement in the *X*, *Y*, and *Z* directions. **(C)** Arm configurations and targets. Participants are cued to reach in the *XY* plane. Reach starts in the “home” sphere and ends in the direction of the “target” sphere. **(D)** Visual feedback. Participants see the avatar arm move in real time along with their movements in the ACT-3D. **(E)** Sample reaching trajectory. **(F)** Sample velocity profile.

Isometric SABD maximum voluntary torques (MVTs) were recorded using a setup composed of a Biodex experimental chair (Biodex Medical Systems, Inc., Shirley, NY, United States) and a six-degree-of-freedom load cell (JR3, Inc., Woodland, CA, United States; model no. 45E15A) as described in previous studies (Dewald and Beer, 2001; Beer et al., 2007). The test arm was secured with a fiberglass cast to the load cell positioned at the distal forearm using delrin plastic rings (Figure 1A). The arm was positioned at 85° SABD, 40° shoulder horizontal flexion, and 90° elbow extension. Using these known joint configurations and Jacobian matrices, forces and moments measured by the load cell were converted to joint torques at the shoulder. Participants were cued to produce maximum isometric SABD torque efforts with five second holds. In order to capture maximum torque production ability, at least three torque efforts within 10% of each other were completed with the last one not being the highest. SABD torques were smoothed using a moving average filter with a 250 ms window immediately following the trial.

Reach kinematics were recorded using the Arm Coordination Training 3D (ACT-3D) which is composed of the admittance controlled HapticMASTER (Moog-FCS BV, Netherlands), a six-degree of freedom load cell end effector (JR3, Inc., Woodland, CA, United States; model no 51E20A4), an instrumented gimbal to record joint angles, and a Biodex experimental chair (Biodex Medical Systems, Inc., Shirley, NY, United States) as described previously (Sukal et al., 2007; Ellis et al., 2008, 2016). The participant’s arm was rigidly attached to the ACT-3D (Figure 1B) and positioned in the same initial configuration as in the isometric setup. Based on the participant’s limb lengths, the reaching target was set near end range of motion (Figure 1C). The participant was cued to complete ballistic reaches with verbal instructions to reach forward as far and as fast as possible with visual feedback provided (Figure 1D). Fingertip position was calculated during movement using inverse kinematics.

Reaching was tested on a haptic frictionless surface (TABLE) and with applied Z forces in the up/downward directions (LIFT). For TABLE trials, the arm was fully supported such that no SABD effort was required and participants could push down on the table without restriction. For LIFT trials, participants generated a SABD torque to abduct the arm from the table and maintained that torque for the duration of the reaching trial. Participants completed 11 trials each of six loading conditions: TABLE and 20, 35, 50, 65, and 80% SABD MVT. The order of the test conditions was randomized for each tested arm to prevent habituation.

### Data Collection and Analysis

Isometric torques were recorded and saved through a data acquisition device (NI-DAQ USB-6225; National Instruments, Austin, TX, United States) at 1000 Hz using customized MATLAB (Mathworks, Inc., Natick, MA, United States) software. Reaching data were recorded and saved at 50 Hz using customized MATLAB software.

#### Reaching Distance

The straight-line distance (*XY* plane) from the starting position to each point in the reaching trajectory was calculated until the maximum reach distance was isolated for each trial. To be included, reach trajectories were required to end within a 40° cone in direction of the target (Figure 1E). For LIFT conditions, only the portion of the trial where the lift was maintained during the reach was considered in analysis. The reach distance deficit (Eq. 1) was calculated for each load level; a larger percentage indicates a greater deficit in reaching ability.

ReachDistanceDeficitLoadLevel=MaxReach-OverallMeanReachLoadLevelMaxReachOverall×100

MaxReach_*Overall*_ is the absolute maximum reach distance across all trials used as the participant-specific functional maximum to compare achievable reaching distance between participants. To capture best performance, MeanReach_*LoadLevel*_ is the average of the top three reach distances for the specified load level. The kurtosis of the top three reach trials for each loading condition was evaluated to ensure that there was not a learning affect in achieving best reaching performance.

#### Fingertip Velocity

The peak velocity of the fingertips was calculated for the top three reach distance trials and averaged together for a single peak velocity for each condition (Figure 1F).

### Statistical Analysis

One-way analyses of variance (ANOVAs) were used to determine if there were differences in age and grip strength ratio between the PH and TD groups. Mann–Whitney *U*-tests were used to determine if there were differences in clinical scores on the FMA-UE, TASC, and ABILHAND/ABILHAND-Kids between the PH injury timing groups. Fisher’s exact tests were used to determine if there were differences in sex and writing hand between the PH and TD groups and in affected arms in the PH injury timing groups. Kruskal–Wallis rank-based non-parametric tests were used to determine whether there were differences in MACS and GMFCS levels between the PH groups. Data were tested for normality using Q–Q plots. Analysis of reach distance deficit and peak velocity was completed with several linear mixed effects models. First, the dominant and non-dominant arms of the TD group were compared using a model with fixed factors of TestArm (two levels, dominant and non-dominant) and Load (six levels, TABLE, 20%MVT, 35%MVT, 50%MVT, 65%MVT, and 80%MVT), and a random factor of participant with an identity covariance structure. Second, reach distance deficit and peak velocity in the three groups were analyzed using linear mixed effects models that included fixed factors of Group (three levels, TD, PRE, PERI), TestArm (two levels, dominant/non-paretic and non-dominant/paretic), and Load (six levels, TABLE, 20%MVT, 35%MVT, 50%MVT, 65%MVT, and 80%MVT) and a random factor of participant with an identity covariance structure. If a significant main effect of TestArm was found, additional linear mixed effects models were computed for each arm independently that included fixed factors of Group (three levels, TD, PRE, PERI) and Load (six levels, TABLE, 20%MVT, 35%MVT, 50%MVT, 65%MVT, and 80%MVT) and a random factor of participant with an identity covariance structure. Finally, for the peak velocity analysis, the PRE and PERI groups were combined into one PH group and another model was computed for each arm independently with fixed factors of Group (two levels, TD, PH) and Load (six levels, TABLE, 20%MVT, 35%MVT, 50%MVT, 65%MVT, and 80%MVT) and a random factor of participant with an identity covariance structure. For all significant main effects and interactions, *post hoc* pairwise comparisons with Bonferroni corrections for multiple comparisons were made. Statistical analysis was completed using SPSS software (version 26, SPSS Inc., Chicago, IL, United States). A value of *p* < 0.05 was considered statistically significant for all tests.

## Results

Thirty-three individuals were consented to participate in this study with complete datasets of both arms tested for 26 participants. Four TD participants completed testing on only one arm due to scheduling constraints or declining to complete testing on the second arm and were excluded from the group analysis. Injury timing was not able to be determined for one participant with PH who was excluded from the dataset. A total of 24 participants were included in the group statistical analysis and were divided into three groups based on previously mentioned criteria: PRE (*n* = 8, mean[SD]: age at testing 11.91[3.45] years, Fugl-Meyer 48.50[15.28]/66), PERI (*n* = 7, mean[SD]: age at testing 12.14[3.96] years, Fugl-Meyer 35.28[7.80]/66), and TD (*n* = 9, mean[SD]: age at testing 10.93[3.87] years). Injury timings were determined by four possible combinations of data: parent report alone (*n* = 4), medical record alone (*n* = 3), parent report and brain imaging (*n* = 7), or medical record and brain imaging (*n* = 1). Two individuals with injury timings after 6 months post full-term were tested as a comparison and are presented in Section “Discussion.” Participant demographics and clinical characteristics are listed in Table 1.

**TABLE 1:** Participant characteristics by Group.

		TD	PRE	PERI	*p*^*a*^	LI^*b*^
Age, mean (SD), years		10.93 (3.87)	11.91 (3.45)	12.14 (3.96)	0.791	17.45 (2.53)
Sex, *n*						
	Male	5	5	4	1.00	2
	Female	4	3	3		0
Grip ratio, mean (SD)^*c*^		0.931 (0.11)	0.513 (0.34)	0.268 (0.17)	<0.001	0 (*n* = 1)
Dominant/non-paretic arm, *n*						
	Right	8	3	1	0.011	1
	Left	1	5	6		1
Arm weight, mean (SD), %^*d*^						
	Dominant	24.07 (5.6)	32.58 (15.3)	22.86 (3.4)	–	29.46 (3.9)
	Non-dominant	24.30 (8.7)	32.52 (8.0)	30.97 (9.7)		36.22 (*n* = 1)
GMFCS, *n*						
	I	NA	4	4	0.648	0
	II	NA	3	3		2
	III	NA	1	0		0
MACS, *n*						
	I	NA	4	1	0.248	0
	II	NA	3	5		0
	III	NA	1	1		2
FMA-UE, mean (SD), x/66		NA	48.50 (15.28)	35.28 (7.80)	0.121	25 (4.24)
TASC-P, mean (SD), x/16		NA	9.40 (2.07)	6.29 (2.56)	0.106	3 (*n* = 1)
TASC-NP, mean (SD), x/16		NA	12.80 (2.77)	13.57 (2.43)	0.530	16 (*n* = 1)
ABL-H, mean (SD), logit		NA	3.65 (2.43)	3.42 (0.89)	1.00	1.89 (0.39)

*^a^P-values reported are the comparisons between TD, PRE, and PERI groups for age, sex, grip ratio, and dominant/non-paretic arms. P-values are comparisons between PRE and PERI for clinical classifications/assessments of GMFCS, MACS, FMA-UE, TASC, and ABL-H. LI group is not included in the statistical analysis. ^b^Individuals tested as late-onset injury comparison but not included in group statistical analyses. ^c^Grip ratio calculated as grip strength in non-dominant/dominant or paretic/non-paretic hand. ^d^Arm weight presented as a percentage of SABD maximum voluntary force as measured in the robotic device: Arm weight (N)/maximum SABD force (N). Abbreviations: ABL-H, ABILHAND-Kids or ABILHAND; FMA-UE, Fugl-Meyer Assessment Upper Extremity; GMFCS, Gross Motor Function Classification System; LI, late-onset injury; MACS, Manual Abilities Classification Scale; MVT, maximum voluntary torque; PERI, PERI-natal; PRE, PRE-natal; SD, standard deviation; SABD, shoulder abduction; TASC-NP, Test of Arm Selective Control non-paretic arm; TASC-P, Test of Arm Selective Control paretic arm; TD, typical development.*

### Comparison of Participant Characteristics and Functional Scores

There was not a significant difference in age [*F*_(2,21)_ = 0.238, *p* = 0.791, η^2^*_*p*_* = 0.022] or sex (*p* = 1.00) in the three participant groups. More individuals in both PH groups wrote with the left hand compared to the TD group (*p* = 0.011). There was a significant difference in grip strength ratio among the three groups [*F*_(2,20)_ = 18.049, *p* < 0.001, η^2^*_*p*_* = 0.643] with *post hoc* testing revealing lower grip strength ratios in the PRE group compared to TD (*p* = 0.004) and in the PERI group compared to TD (*p* < 0.001). A lower grip strength ratio indicates greater asymmetry between the paretic and non-paretic hands with the paretic hand being weaker. There was no significant difference in grip strength ratio in the PRE and PERI groups (*p* = 0.164).

The right arm was the paretic arm for the majority of individuals with PH with no significant difference detected between the two injury timing groups (*p* = 0.569). Manual ability and gross motor classifications between injury timing groups were similar with no significant differences found between groups for MACS (*H* = 1.335, *p* = 0.248, asymptotic two-sided) or GMFCS (*H* = 0.208, *p* = 0.648, asymptotic two-sided) levels. Similarly, there were no significant differences found between injury timing groups for scores on the remaining clinical assessments of FMA-UE (*U* = 14.5, *p* = 0.121, exact two-sided), TASC:Paretic (*U* = 7.0, *p* = 0.106, exact two-sided), TASC:Non-paretic (*U* = 22.0, *p* = 0.530, exact two-sided) or ABILHAND-Kids (*U* = 28.0, *p* = 1.00, exact two-sided). Based on the information captured from these clinical assessments, individuals in the PRE and PERI groups had similar impairment levels and clinical presentations.

### Kinematic Findings

Reach distance deficit and peak velocity were compared as a function of group, test arm, and loading condition to evaluate the effect of modulating shoulder effort and of injury timing on reaching kinematics. Group results can be found in Table 2 and main effect and interaction results from linear mixed effects models can be found in Table 3.

**TABLE 2:** Mean (SD) of reaching kinematics by Group.

	Reach distance deficit			Fingertip velocity (m/s)		
	TD	PRE	PERI	TD	PRE	PERI
**Table**						
D/NP	0.0624 (0.042)	0.0585 (0.059)	0.0624 (0.048)	1.5102 (0.334)	1.1042 (0.281)	1.5532 (0.448)
ND/P	0.0607 (0.033)	0.0785 (0.066)	0.0368 (0.020)	1.4531 (0.299)	1.0620 (0.407)	1.4078 (0.238)
**20%MVT**						
D/NP	0.0817 (0.038)	0.1057 (0.033)	0.0785 (0.038)	1.3447 (0.338)	1.0221 (0.463)	1.3467 (0.451)
ND/P	0.0681 (0.029)	0.1027 (0.085)	0.1014 (0.070)	1.3833 (0.339)	0.9947 (0.566)	1.1664 (0.282)
**35%MVT**						
D/NP	0.0869 (0.059)	0.0882 (0.044)	0.0909 (0.026)	1.4693 (0.381)	1.1697 (0.530)	1.4297 (0.526)
ND/P	0.1159 (0.049)	0.1016 (0.073)	0.1245 (0.095)	1.3855 (0.323)	1.1048 (0.355)	1.0268 (0.359)
**50%MVT**						
D/NP	0.0918 (0.049)	0.1269 (0.059)	0.1208 (0.058)	1.4499 (0.367)	1.1216 (0.482)	1.4127 (0.408)
ND/P	0.1266 (0.054)	0.1214 (0.051)	0.1521 (0.086)	1.2955 (0.418)	0.9691 (0.397)	0.9055 (0.288)
**65%MVT**						
D/NP	0.1010 (0.051)	0.1272 (0.070)	0.1110 (0.084)	1.4578 (0.365)	1.1015 (0.535)	1.4244 (0.394)
ND/P	0.1356 (0.055)	0.1537 (0.098)	0.1600 (0.113)	1.3444 (0.421)	0.9069 (0.448)	0.9731 (0.313)
**80%MVT**						
D/NP	0.1215 (0.042)	0.2087 (0.160)	0.1522 (0.057)	1.3852 (0.479)	1.0465 (0.416)	1.1015 (0.477)
ND/P	0.1781 (0.093)	0.2028 (0.117)	0.2512 (0.193)	1.2637 (0.507)	0.8738 (0.322)	0.9253 (0.238)

*Group mean values with standard deviations for reach distance deficit and fingertip velocity for each group and arm. Reach distance deficit presented as a value from 0 to 1 where 0 indicates no deficit in reaching performance and 1 indicates complete deficit. Abbreviations: D, dominant; MVT, maximum voluntary torque; ND, non-dominant; NP, non-paretic; P, paretic; PERI, PERI-natal; PRE, PRE-natal; SD, standard deviation; TD, typical development.*

**TABLE 3:** Main effect and interaction results from linear mixed models.

	Main effects			Interactions			
	Group	TestArm	Load	Group × TestArm	Group × Load	Load × TestArm	Group × TestArm × Load
**TD only**							
*Reach deficit*	–	ns	***	–	–	ns	–
*Velocity*	–	ns	ns	–	–	ns	–
**Three groups^*a*^**							
*Reach deficit*							
Both arms	ns	ns	***	ns	ns	ns	ns
*Velocity*							
Both arms	ns	*	***	ns	*	*	ns
ND-P arm	ns	–	***	–	ns	–	–
D-NP arm	ns	–	***	–	ns	–	–
**Two groups^*b*^**							
*Velocity*							
ND-P arm	*	–	***	–	ns	–	–
D-NP arm	ns	–	*	–	ns	–	–

^a^Analysis of TD, PRE, and PERI groups. ^b^Analysis of TD and PH groups when PH is the combination of the PRE and PERI groups. Reach distance deficit and peak velocity were analyzed with linear mixed effects models with factors Group, TestArm, and Load. Asterisks indicate significant effects at p < 0.05 (*) and p < 0.001 (***). ns indicates p > 0.05; exact p-values can be found in the text. Abbreviations: D, dominant; ND, non-dominant; NP, non-paretic; ns, non-significant; P, paretic; PERI, PERI-natal; PRE, PRE-natal; TD, typical development.

#### TD: Reaching and Velocity—Both Arms

Individuals in the TD group demonstrated similar performance between the dominant and non-dominant arms for both reaching and velocity. For reach distance deficit, there was a significant main effect of Load [*F*_(5,80)_ = 9.323, *p* < 0.001] but not TestArm [*F*_(1,8)_ = 2.759, *p* = 0.135] or the interaction of TestArm and Load [*F*_(5,80)_ = 1.593, *p* = 0.172]. *Post hoc* comparisons showed that individuals in the TD group reached further in the table supported condition compared to 50%MVT (*p* = 0.024), 65%MVT (*p* = 0.003), and 80%MVT (*p* < 0.001). For peak fingertip velocity, there was not a significant main effect of Load [*F*_(5,80)_ = 1.663, *p* = 0.153], TestArm [*F*_(__1__,8)_ = 0.845, *p* = 0.385], or the interaction of TestArm and Load [*F*_(5,80)_ = 0.635, *p* = 0.674].

#### Reach Distance Deficit

##### TD compared to PRE and PERI—Both arms

**FIGURE 2:**
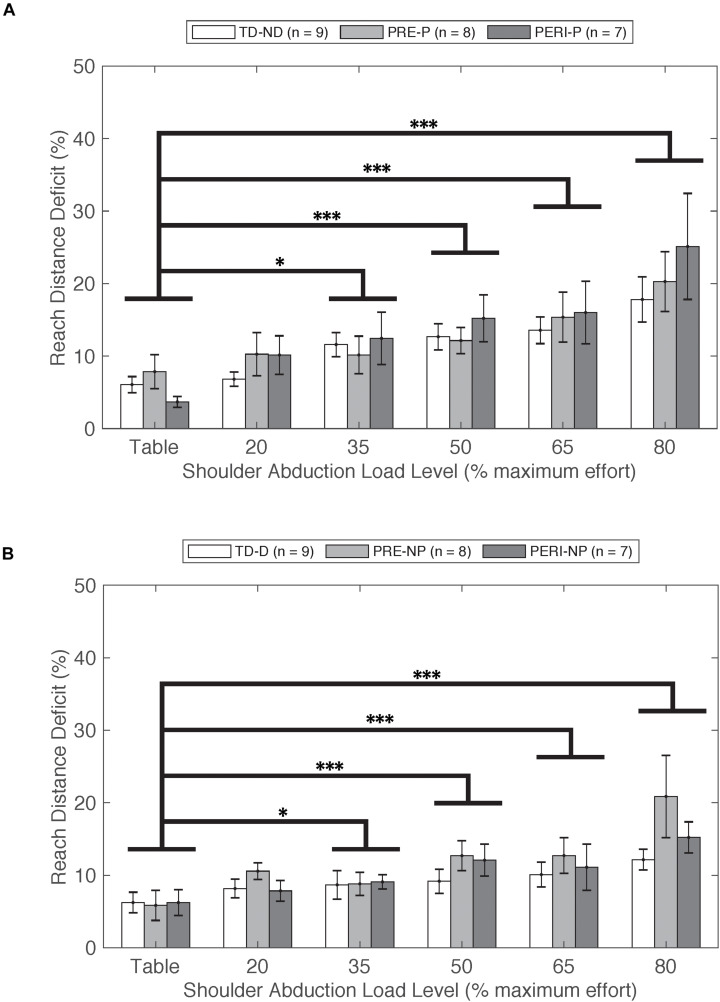
Reach distance deficit as a function of SABD level compared between three groups for **(A)** non-dominant (TD) and paretic arms (PRE/PERI) and **(B)** dominant (TD) and non-paretic arms (PRE/PERI). Reach distance deficit was calculated based on Eq. 1. In the group analysis for both arms and all groups, a significant main effect of Load [*F*_(5,210)_ = 22.339, *p* < 0.001] was detected indicating that increased drive to the shoulder resulted in decreased reaching ability in all three groups. Abbreviations: D, dominant; P, paretic; PERI, PERI-natal; PRE, PRE-natal; ND, non-dominant; NP, non-paretic; SABD, shoulder abduction; TD, typical development. Error bars ± standard error. ^∗^*p* < 0.05 and ^∗∗∗^*p* < 0.001.

Reach distance deficits for all groups and arms can be seen in Figure 2. Reach distance decreased with increasing SABD load for all groups reaching with both arms. There was a significant main effect of Load [*F*_(5,210)_ = 22.339, *p* < 0.001]. *Post hoc* comparisons with Bonferroni corrections for multiple comparison revealed that participants reached further in the table supported condition compared to 35%MVT (*p* = 0.022), 50%MVT (*p* < 0.001), 65%MVT (*p* < 0.001), and 80%MVT (*p* < 0.001). As an example, when reaching with the non-dominant or paretic arm, participants on average demonstrated an 11.7% (TD), 12.4% (PRE), or 21.4% (PERI) decrease in reaching distance at 80%MVT compared to the table condition (Table 2). Other main effects and interactions were not significant {Group [*F*_(2,21)_ = 0.710, *p* = 0.503]; TestArm [*F*_(1,21)_ = 3.717, *p* = 0.067]; Group × TestArm [*F*_(__2,21)_ = 0.465, *p* = 0.635]; Group × Load [*F*_(10,210)_ = 0.758, *p* = 0.669]; Load × TestArm [*F*_(5,210)_ = 1.217, *p* = 0.302]; Group × TestArm × Load [*F*_(10,210)_ = 0.636, *p* = 0.782]}. These results demonstrate that all three groups performed similarly in their reaching distance regardless of which arm was reaching.

#### Peak Fingertip Velocity

##### TD compared to PRE and PERI—Both arms

**FIGURE 3:**
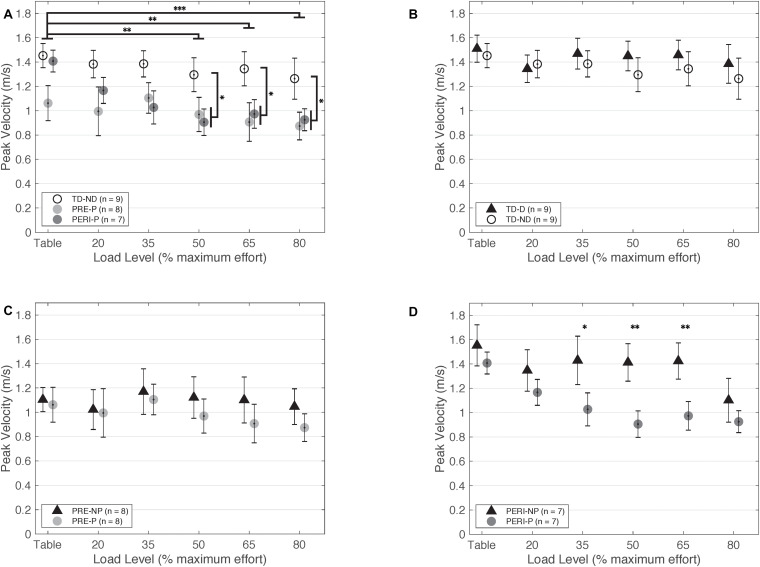
Peak fingertip velocity as a function of SABD load level. **(A)** Non-dominant/paretic arm for all groups. Significant main effects of Load [*F*_(5,110)_ = 5.824, *p* < 0.001] and Group [TD or PH; *F*_(1,22)_ = 5.516, *p* = 0.028] were found. Individuals with PH achieved lower peak velocities in the paretic arm compared to the TD group reaching with the non-dominant arm. **(B)** Dominant and non-dominant arms for TD group. **(C)** Non-paretic and paretic arms for PRE group. **(D)** Non-paretic and paretic arms for PERI group. Significant pairwise comparisons were found for 35%MVT, 50%MVT, and 65%MVT indicating lower peak velocities in the paretic arm compared to the non-paretic arm in this group. Abbreviations: D, dominant; P, paretic; PERI, PERI-natal; PRE, PRE-natal; ND, non-dominant; NP, non-paretic; SABD, shoulder abduction; TD, typical development. Error bars ± standard error. *p < 0.05, **p < 0.01, ***p < 0.001.

Comparative results of peak fingertip velocity can be seen in Figure 3. When comparing peak fingertip velocity between groups, there was a significant main effect of Load [*F*_(5,210)_ = 9.316, *p* < 0.001] and TestArm [*F*_(__1,21)_ = 5.696, *p* = 0.026]. *Post hoc* pairwise comparisons of Load with Bonferroni corrections for multiple comparisons revealed significant differences in velocity between TABLE and 20%MVT (*p* = 0.006), 50%MVT (*p* = 0.001), 65%MVT (*p* = 0.002), and 80%MVT (*p* < 0.001) and between 80%MVT and 35%MVT (*p* < 0.001). Participants in all three groups demonstrated a significant decrease in peak velocity at higher SABD load levels. For example, when reaching with the non-dominant or paretic arm, participants on average demonstrated a 0.189 m/s (TD), 0.188 m/s (PRE), or 0.435 m/s (PERI) decrease in peak velocity at 80%MVT compared to the table condition (Table 2). *Post hoc* pairwise comparisons for TestArm with Bonferroni corrections for multiple comparisons revealed significant differences between arms in the PERI group for loads 35%MVT (*p* = 0.014), 50%MVT (*p* = 0.002), and 65%MVT (*p* = 0.006) indicating velocity asymmetry in the PERI group that was not present in the TD and PRE groups (Figure 3B). Additionally, significant interactions were found for Group × Load [*F*_(10,210)_ = 2.069, *p* = 0.028] and TestArm × Load [*F*_(5,210)_ = 2.601, *p* = 0.026]. Other main effects and interactions were not significant {Group [*F*_(2,21)_ = 2.554, *p* = 0.102]; Group × TestArm [*F*_(2,21)_ = 1.005, *p* = 0.383]; Group × TestArm × Load [*F*_(10,210)_ = 0.686, *p* = 0.737]}. Because the main effect of TestArm was found to be significant, the dominant/non-paretic and non-dominant/paretic arms were tested independently by computing linear mixed models with fixed factors of Group and Load and random factor of participant.

##### TD compared to PRE and PERI—Non-dominant/paretic arm

When comparing just the non-dominant/paretic arms, there was a significant main effect of Load [*F*_(5,105)_ = 8.460, *p* < 0.001] on velocity performance. *Post hoc* pairwise comparisons for Load with Bonferroni corrections for multiple comparisons revealed significant differences between TABLE and 50%MVT (*p* < 0.001), 65%MVT (*p* < 0.001), and 80%MVT (*p* < 0.001) and between 80%MVT and 20%MVT (*p* = 0.035) when comparing all three groups indicating a decrease in velocity with increased load level. Other main effects and interactions were not significant {Group [*F*_(2,21)_ = 2.769, *p* = 0.086]; Group × Load [*F*_(10,105)_ = 1.819, *p* = 0.066]}. The model was adjusted to compare the PRE and PERI groups as one group to the TD group. There was a significant main effect of Load [*F*_(5,110)_ = 5.824, *p* < 0.001] and Group [*F*_(1,22)_ = 5.516, *p* = 0.028] but not the interaction of Group × Load [*F*_(5,110)_ = 0.607, *p* = 0.695]. *Post hoc* comparisons for Load with Bonferroni corrections for multiple comparisons revealed significant differences between TABLE and 50%MVT (*p* = 0.002), 65%MVT (*p* = 0.008), and 80%MVT (*p* < 0.001) for all groups. Additional *post hoc* comparisons revealed significant differences between TD and PH for 50%MVT (*p* = 0.031), 65%MVT (*p* = 0.014), and 80%MVT (*p* = 0.027) as shown in Figure 3A.

##### TD compared to PRE and PERI—Dominant/non-paretic arm

When comparing just the dominant/non-paretic arms, there was a significant main effect of Load [*F*_(5,105)_ = 3.90, *p* = 0.003] on velocity performance. *Post hoc* pairwise comparisons for Load with Bonferroni corrections for multiple comparisons revealed significant differences between TABLE and 80%MVT (*p* = 0.005) and 80%MVT and 35%MVT (*p* = 0.033) when comparing all three groups. These results indicate a decrease in velocity at the higher load levels when reaching with the dominant or non-paretic arm. Other main effects and interactions were not significant {Group [*F*_(2,21)_ = 1.798, *p* = 0.190]; Group × Load [*F*_(10,105)_ = 1.014, *p* = 0.437]}. The model was adjusted to compare the PRE and PERI groups together to the TD group. There was still a significant main effect of Load [*F*_(5,110)_ = 2.935, *p* = 0.016] with *post hoc* pairwise comparisons revealing significant differences between TABLE and 80%MVT (*p* = 0.037). Other main effects and interactions were not significant {Group [*F*_(1,22)_ = 1.553, *p* = 0.226]; Group × Load [*F*_(5,110)_ = 0.390, *p* = 0.855]}.

### Summary of Results

In summary, SABD load was found to have a significant effect on both reaching performance and peak fingertip velocity for all three tested groups. TestArm (dominant/non-paretic or non-dominant/paretic) and Group (TD or PH) had additional significant main effects on peak fingertip velocity.

## Discussion

The goal of this study was to evaluate the presence of the flexion synergy in the paretic upper extremity during a ballistic reach in individuals with PH from early-onset brain injuries. We used a haptic robotic device that allowed us to systematically modulate the SABD torque required during the task and quantify changes in reaching distance and peak velocity. This work evaluates reaching performance in participants age 6–17 years old with and without neurological impairment while challenging the nervous system to generate SABD loads up to 80%MVT. We found that individuals with early-onset PH demonstrate similar performance in reaching distance as controls without neurological impairment and there was no difference between arms. When lifting loads of 35%MVT and greater, we found a statistically significant decrease in reaching ability in the combined analysis of all groups and arms, indicating that there was a relative cost for all participants to add an abduction torque load to the task. In addition, these data show that the presence of an early-onset brain injury does not have a differential impact on ability to individually control the joints of the shoulder and elbow even as the demand for remaining corticospinal resources to activate the shoulder abductors increases. When comparing peak velocity achieved, we found that individuals in the PRE and PERI groups achieved lower peak velocities in the paretic arm compared to controls at most load levels. These results highlight that for this specific reaching task, individuals with PH experience a relative reduction in the ability to generate ballistic movements rather than losses of independent joint control impacting reaching distance. The underlying neural mechanisms enabling maintained independent joint control in individuals with early-onset lesions could be related to a relative preservation of connections from corticospinal motor pathways due to the time of lesion.

### Neural Circuitry Preserved in Early Injury

Participants with early-onset lesions did not perform statistically different from participants without neurological impairment in reaching distance across all load levels. The maintenance of independent joint control in children and adolescents with early-onset brain lesions may be indicative of the neural connections present for descending motor control. Injury to the developing brain leads to specific patterns of (re)organization based on timing of injury (Martin, 2005; Jaspers et al., 2015). The three proposed patterns of CST wiring after early lesions include ipsilateral, bilateral, and contralateral (Jaspers et al., 2015). In the earlier stages of development (24–34 weeks of gestation), the most common lesions occur in the periventricular white matter leading to a decrease in the competitive withdrawal and maintenance of the ipsilateral CST (Jaspers et al., 2015). In weeks 34–38 of gestation through the first 28 days post full-term, blood flow has migrated to cortical and subcortical regions increasing the likelihood of lesions occurring in these areas (Jaspers et al., 2015). These lesions may leave the crossed CST from the lesioned hemisphere partially intact which could minimize the interruption of typical pruning processes (Staudt, 2010; Jaspers et al., 2015). The injury timings in our cohort align with the periods of development suggested to allow a maintenance of ipsilateral CST connections from the non-lesioned hemisphere, a partially intact CST from the lesioned hemisphere, or connections from both hemispheres. The presence of direct connections from the cortex to the spinal cord has been investigated previously using transcranial magnetic stimulation (TMS) in groups that experienced different injury timings. Individuals with periventricular white matter lesions are most similar in timing to our PRE cohort. Simon-Martinez et al. and Mailleux et al. demonstrated that 31% (Simon-Martinez et al., 2018) to 38% (Mailleux et al., 2020) of individuals with periventricular white matter lesions in their cohort had ipsilateral CST connections and 23% (Simon-Martinez et al., 2018) to 24% (Mailleux et al., 2020) had bilateral CST connections. Individuals with cortico-subcortical lesions are most similar in timing to our PERI cohort. In those same studies, 33% (Mailleux et al., 2020) to 44% (Simon-Martinez et al., 2018) of individuals with cortico-subcortical lesions had ipsilateral CST connections while 44% (Simon-Martinez et al., 2018) to 55% (Mailleux et al., 2020) had bilateral CST connections. The remaining individuals had contralateral CST connections. The presence of ipsilateral, bilateral, or contralateral CST pathways found even in individuals with large lesions indicates a resilience of the developing brain not afforded to individuals who sustain a stroke in adulthood. Findings from individuals with early injuries in our cohort that demonstrate a maintained ability to reach even as the SABD demand increases align with the hypothesis of relative preservation of CST pathways.

### Neural Mechanisms Implicated in Late-Onset PH and Adult-Onset Hemiplegia

**FIGURE 4:**
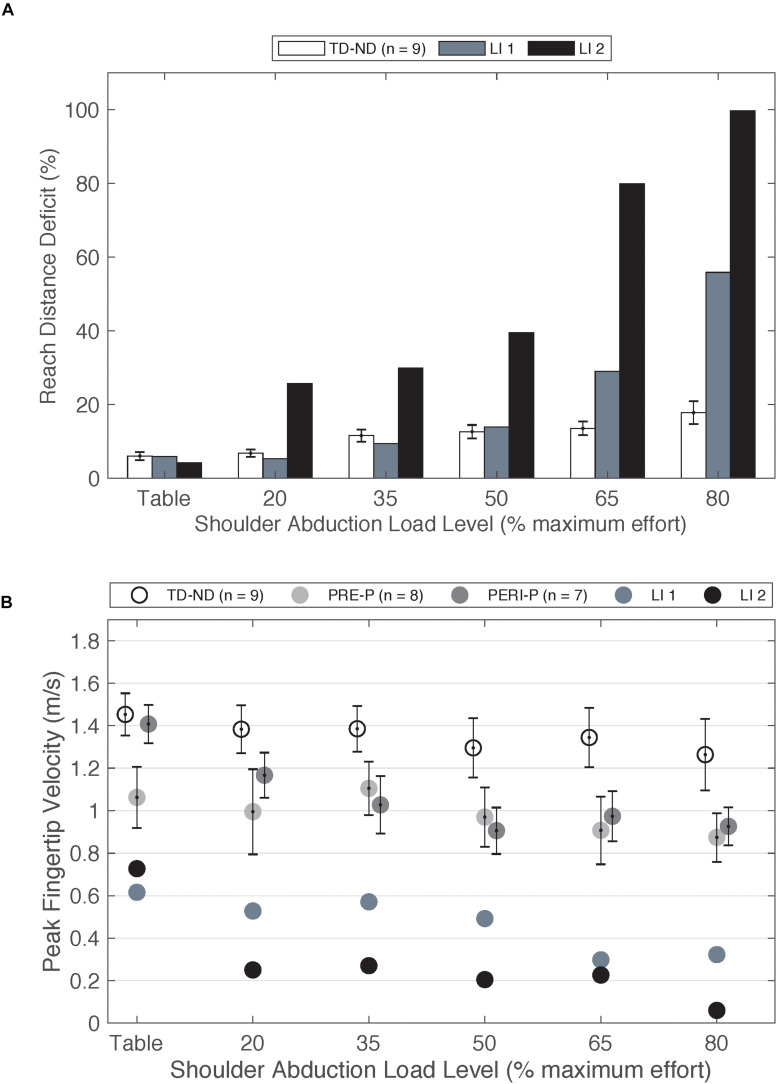
**(A)** Reach distance deficit of the non-dominant arm of the TD group compared to paretic arm of two individuals with late-onset injuries. **(B)** Peak fingertip velocity of the non-dominant arm of TD group and paretic arm of the PRE and PERI groups compared to the paretic arms of two individuals with late-onset injuries. Abbreviations: LI, late-onset injury; P, paretic; PERI, PERI-natal; PRE, PRE-natal; ND, non-dominant; TD, typical development. Error bars ± standard error.

As a comparison to individuals with injuries prior to 6 months, two individuals fitting the original inclusion criteria who sustained strokes at ages 8 and 9 years old were also tested (*n* = 2, mean[SD]: age at testing 17.45[2.53] years, Fugl-Meyer 29.46[3.9]/66) but not included in the group statistical analysis due to the low sample size. Injury timing after 6 months post full-term was determined by medical record and brain imaging (*n* = 1) and parent report and brain imaging (*n* = 1). In contrast to early-onset PH, these individuals show large deficits in reaching with increased load (Figure 4A) when compared to individuals in the TD group. Furthermore, the peak velocity achieved is less than half that of the PRE and PERI groups (Figure 4B). These findings are similar to other studies in adult-onset hemiplegia investigating reaching area (Sukal et al., 2007), elbow joint angular velocity (Ellis et al., 2017), and reaching distance (McPherson et al., 2018) which demonstrated significant decreases in performance as the SABD load is increased.

Trends from these two participants are very closely aligned with previous work in adult-onset hemiplegia, a population that demonstrates large expressions of the flexion synergy (Sukal et al., 2007; Ellis et al., 2017; McPherson et al., 2018). Different available neural resources for paretic arm control is one possible explanation for differences in performance on upper extremity reaching tasks between early-onset PH, late-onset PH, and adult-onset hemiplegia. Contralateral and ipsilateral cortical connections have been investigated in adults post stroke using TMS approaches (Netz et al., 1997; Schwerin et al., 2008) including a paired pulse approach (Schwerin et al., 2011) to enhance the ability to elicit motor evoked potentials (MEPs) in moderate to severely impaired individuals. Netz et al. (1997) investigated the response of TMS to the non-lesioned hemisphere on a cohort of adults post stroke and found longer latencies of ipsilateral MEPs compared to the contralateral MEPs. One hypothesized explanation for the increased latency of these responses was the activation of multi-synaptic cortico-reticulospinal pathways (Netz et al., 1997). McPherson et al. (2018) highlighted that an increased reliance on cortico-reticulospinal pathways, in the absence of viable corticospinal connections, may explain the loss of selective control of the upper extremity. The cortico-reticulospinal tract branches into multiple muscle groups compared to the direct CST (Jankowska and Edgley, 2006; Yeo et al., 2012). Therefore, the ability to selectively control joints in the paretic arm is diminished. Furthermore, as the demand for neural resources increases as a function of SABD load level, there is a decreased ability to isolate purposeful movement in the shoulder from movement in the elbow (Miller and Dewald, 2012; Ellis et al., 2016). The previous work in adult-onset hemiplegia in combination with these two late-onset PH cases highlight the differences the loss of upper extremity individual joint control between early- and late-onset hemiplegia.

### Decreased Peak Velocity in PH Groups

Participants were cued to reach as fast as possible toward the target for all trials. We found that while there was a relative maintenance in reaching distance between groups, individuals with PH achieved significantly lower peak velocities in the paretic arm compared to the non-dominant arm of the TD group. This decrease in peak velocity in the paretic arm is indicative of impairment that could be attributed to biomechanical coupling or impairment to control that is not present in the TD cohort. Interestingly, individuals in the TD and PRE groups achieved similar peak velocities between arms while individuals in the PERI group achieved velocities close to the PRE group in the paretic arm and close to the TD group in the non-paretic arm. While the ipsilateral CST projections might be available for use, they may not be as efficient as contralateral projections (Jaspers et al., 2015), resulting in measurable impairment in performance. Although this study was not designed to investigate specific mechanisms of movement speed reductions, findings are aligned with other studies that have found decreased movement speed in individuals with hemiplegic cerebral palsy (Jaspers et al., 2009). Potential mechanisms that could be involved in reductions in movement speed that should be explored further include the impact of bone and muscle development, the ability to fully activate muscles selected for a motor task due to a relative loss of corticospinal resources, and coordination for completing a novel task.

### Clinical and Scientific Significance

There has been a wealth of information about the motor control of adults with hemiplegia including the pervasive influence of flexion patterns in the upper extremity likely mediated by bulbospinal pathways (Sukal et al., 2007; Lan et al., 2017; McPherson et al., 2018; McPherson and Dewald, 2019). In contrast, we have shown minimal expression of the flexion synergy in individuals with early-onset PH even at high levels of SABD loading. Although the flexion synergy is not a primary challenge, individuals with PH do demonstrate functional impairments that limit fine motor control necessary for daily activities (Holmefur et al., 2010). Selective control of the hand is more exclusively controlled by the CST; therefore, hand functionality is likely to be more severely impacted after injury (Lawrence and Kuypers, 1968). With a partially intact CST, clinicians can focus on rehabilitation activities to upregulate remaining projections (Hung et al., 2017; Bleyenheuft et al., 2020). This may be especially effective during early years of life prior to pruning or reinforcement of aberrant connections from the cortex to the muscles and has the theoretical potential to maximize strength and coordination of the hand (Basu, 2014). Quantitative metrics are being developed to explore hand control in this population (Hill and Dewald, 2018) and to address rehabilitative needs of the fingers (McCall et al., 2019).

### Limitations

We tested our participants in a stable, supported sitting position for all tasks. Observationally, several individuals in our PRE and PERI injury timing groups demonstrated associated movements or movement overflow to the non-tested arm and one or both legs during higher SABD load tasks. Participants were not cued to suppress any of these movements. However, it is possible that the increased use of corticospinal resources required to control the legs during a higher effort walking task could diminish the remaining resources to control the paretic arm and increase the influence of indirect cortico-reticulospinal pathways thus resulting in the expression of the upper extremity flexion synergy (Galli et al., 2012; Hung and Meredith, 2014).

Due to the range of experiences with therapeutic interventions available to different individuals prior to participation in this study, we did not control for the type or amount of past therapeutic/activity-based intervention for our study cohort. Additionally, the study sample size is small due to considerable challenges in recruitment of a scientifically homogeneous group required for drawing conclusions about underlying neurological mechanisms.

## Conclusion

Upper extremity function continues to be a leading focus of therapeutic intervention for individuals with PH regardless of injury timing. We have shown that individuals with early lesions are minimally influenced by the particular impairment of the flexion synergy during a high effort reaching task. Findings indicate a distinction in the underlying neural resources still available after an early compared to later unilateral brain injury. Additional research investigating losses in hand dexterity will elucidate the extent to which a partially intact CST impacts distal joints in this population.

## Data Availability Statement

The raw data supporting the conclusions of this article will be made available by the authors, without undue reservation.

## Ethics Statement

The studies involving human participants were reviewed and approved by the Institutional Review Board at Northwestern University. Written informed consent to participate in this study was provided by the participants’ legal guardian/next of kin.

## Author Contributions

Both authors contributed to the conception and design of the work, contributed to the interpretation of data, editing the manuscript, and approving the submitted version, and agreed to be accountable for all aspects of the work. NH contributed to data acquisition, data analysis, and initial manuscript preparation.

## Conflict of Interest

The authors declare that the research was conducted in the absence of any commercial or financial relationships that could be construed as a potential conflict of interest.

## References

[B1] Arnould C. Penta M. Renders A. Thonnard J. L. (2004). ABILHAND-Kids–a measure of manual ability in children with cerebral palsy. *Neurology* 63 1045–1052. 10.1212/01.Wnl.0000138423.77640.37 15452296

[B2] Basu A. P. (2014). Early intervention after perinatal stroke: opportunities and challenges. *Dev. Med. Child Neurol.* 56 516–521. 10.1111/dmcn.12407 24528276 PMC4020312

[B3] Beer R. F. Ellis M. D. Holubar B. G. Dewald J. P. (2007). Impact of gravity loading on post-stroke reaching and its relationship to weakness. *Muscle Nerve* 36 242–250. 10.1002/mus.20817 17486581 PMC2866301

[B4] Beer R. F. Given J. D. Dewald J. P. (1999). Task-dependent weakness at the elbow in patients with hemiparesis. *Arch. Phys. Med. Rehabil.* 80 766–772. 10.1016/s0003-9993(99)90225-3 10414760

[B5] Bleyenheuft Y. Dricot L. Ebner-Karestinos D. Paradis J. Saussez G. Renders A. (2020). Motor skill training may restore impaired corticospinal tract fibers in children with cerebral palsy. *Neurorehabil. Neural Repair* 34 533–546. 10.1177/1545968320918841 32407247

[B6] Broeks J. G. Lankhorst G. J. Rumping K. Prevo A. J. (1999). The long-term outcome of arm function after stroke: results of a follow-up study. *Disabil. Rehabil.* 21 357–364. 10.1080/096382899297459 10503976

[B7] Brunnstrom S. (1970). *Movement Therapy in Hemiplegia: a Neurophysiological Approach.* Hagerstown, MD: Medical Dept. Harper & Row.

[B8] Cahill-Rowley K. Rose J. (2014). Etiology of impaired selective motor control: emerging evidence and its implications for research and treatment in cerebral palsy. *Dev. Med. Child Neurol.* 56 522–528. 10.1111/dmcn.12355 24359183

[B9] Dewald J. P. Beer R. F. (2001). Abnormal joint torque patterns in the paretic upper limb of subjects with hemiparesis. *Muscle Nerve* 24 273–283. 11180211 10.1002/1097-4598(200102)24:2<273::aid-mus130>3.0.co;2-z

[B10] Dewald J. P. Pope P. S. Given J. D. Buchanan T. S. Rymer W. Z. (1995). Abnormal muscle coactivation patterns during isometric torque generation at the elbow and shoulder in hemiparetic subjects. *Brain* 118(Pt 2) 495–510. 10.1093/brain/118.2.495 7735890

[B11] Dewald J. P. Sheshadri V. Dawson M. L. Beer R. F. (2001). Upper-limb discoordination in hemiparetic stroke: implications for neurorehabilitation. *Top. Stroke Rehabil.* 8 1–12. 10.1310/WA7K-NGDF-NHKK-JAGD 14523747

[B12] Eliasson A. C. Krumlinde-Sundholm L. Rosblad B. Beckung E. Arner M. Ohrvall A. M. (2006). The manual ability classification system (MACS) for children with cerebral palsy: scale development and evidence of validity and reliability. *Dev. Med. Child Neurol.* 48 549–554. 10.1017/S0012162206001162 16780622

[B13] Ellis M. D. Lan Y. Yao J. Dewald J. P. (2016). Robotic quantification of upper extremity loss of independent joint control or flexion synergy in individuals with hemiparetic stroke: a review of paradigms addressing the effects of shoulder abduction loading. *J. Neuroeng. Rehabil.* 13:95. 10.1186/s12984-016-0203-0 27794362 PMC5086410

[B14] Ellis M. D. Schut I. Dewald J. P. A. (2017). Flexion synergy overshadows flexor spasticity during reaching in chronic moderate to severe hemiparetic stroke. *Clin. Neurophysiol.* 128 1308–1314. 10.1016/j.clinph.2017.04.028 28558314 PMC5507628

[B15] Ellis M. D. Sukal T. DeMott T. Dewald J. P. (2008). Augmenting clinical evaluation of hemiparetic arm movement with a laboratory-based quantitative measurement of kinematics as a function of limb loading. *Neurorehabil. Neural Repair* 22 321–329. 10.1177/1545968307313509 18326888 PMC2826208

[B16] Eyre J. A. Taylor J. P. Villagra F. Smith M. Miller S. (2001). Evidence of activity-dependent withdrawal of corticospinal projections during human development. *Neurology* 57 1543–1554. 11706088 10.1212/wnl.57.9.1543

[B17] Fasoli S. Fragala-Pinkham M. Haley S. (2009). “Fugl-meyer assessment: reliability for children with hemiplegia,” in *Proceedings of the ACRM-ASNR Joint Educational Conference*, (Archives of Physical Medicine and Rehabilitation), Denver, CO.

[B18] Fugl-Meyer A. R. Jaasko L. Leyman I. Olsson S. Steglind S. (1975). The post-stroke hemiplegic patient. 1. a method for evaluation of physical performance. *Scand. J. Rehabil. Med.* 7 13–31. 1135616

[B19] Galli M. Cimolin V. Crivellini M. Romkes J. Albertini G. Brunner R. (2012). Quantification of upper limb motion during gait in children with hemiplegic cerebral palsy. *J. Dev. Phys. Disabil.* 24 1–8. 10.1007/s10882-011-9250-4

[B20] Hill N. M. Dewald J. P. A. (2018). Development of a method to quantify abnormal kinetic and kinematic coupling patterns during functional movements in the paretic arm and hand of individuals with pediatric hemiplegia. *Conf. Proc. IEEE Eng. Med. Biol. Soc.* 2018 2280–2283. 10.1109/EMBC.2018.8512841 30440861 PMC7211269

[B21] Holmefur M. Krumlinde-Sundholm L. Bergstrom J. Eliasson A. C. (2010). Longitudinal development of hand function in children with unilateral cerebral palsy. *Dev. Med. Child Neurol.* 52 352–357. 10.1111/j.1469-8749.2009.03364.x 19583744

[B22] Hung Y. C. Brandao M. B. Gordon A. M. (2017). Structured skill practice during intensive bimanual training leads to better trunk and arm control than unstructured practice in children with unilateral spastic cerebral palsy. *Res. Dev. Disabil.* 60 65–76. 10.1016/j.ridd.2016.11.012 27912104

[B23] Hung Y. C. Meredith G. S. (2014). Influence of dual task constraints on gait performance and bimanual coordination during walking in children with unilateral Cerebral Palsy. *Res. Dev. Disabil.* 35 755–760. 10.1016/j.ridd.2014.01.024 24529863

[B24] Hunt R. W. Inder T. E. (2006). Perinatal and neonatal ischaemic stroke: a review. *Thromb. Res.* 118 39–48. 10.1016/j.thromres.2004.12.021 16709474

[B25] Hurley D. S. Sukal-Moulton T. Msall M. E. Gaebler-Spira D. Krosschell K. J. Dewald J. P. (2011). The cerebral palsy research registry: development and progress toward national collaboration in the United States. *J. Child Neurol.* 26 1534–1541. 10.1177/0883073811408903 21677201 PMC3223319

[B26] Jankowska E. Edgley S. A. (2006). How can corticospinal tract neurons contribute to ipsilateral movements? A question with implications for recovery of motor functions. *Neuroscientist* 12 67–79. 10.1177/1073858405283392 16394194 PMC1890027

[B27] Jaspers E. Byblow W. D. Feys H. Wenderoth N. (2015). The corticospinal tract: a biomarker to categorize upper limb functional potential in unilateral cerebral palsy. *Front. Pediatr.* 3:112. 10.3389/fped.2015.00112 26779464 PMC4701904

[B28] Jaspers E. Desloovere K. Bruyninckx H. Molenaers G. Klingels K. Feys H. (2009). Review of quantitative measurements of upper limb movements in hemiplegic cerebral palsy. *Gait Posture* 30 395–404. 10.1016/j.gaitpost.2009.07.110 19679479

[B29] Koman L. A. Smith B. P. Williams R. Richardson R. Naughton M. Griffin L. (2013). Upper extremity spasticity in children with cerebral palsy: a randomized, double-blind, placebo-controlled study of the short-term outcomes of treatment with botulinum A toxin. *J. Hand Surg. Am.* 38 435–446.e1. 10.1016/j.jhsa.2012.12.019 23428186

[B30] Krosschell K. Moulton T. Barnum J. Block K. Nichols A. Salzman E. (2015). “Reliability of the test of arm selective control (TASC),” in *Proceedings of the 69th Annual Meeting of the American Academy for Cerebral Palsy and Developmental Medicine*, (Developmental Medicine and Child Neurology), Austin, TX.

[B31] Kuczynski A. M. Dukelow S. P. Hodge J. A. Carlson H. L. Lebel C. Semrau J. A. (2018a). Corticospinal tract diffusion properties and robotic visually guided reaching in children with hemiparetic cerebral palsy. *Hum. Brain Mapp.* 39 1130–1144. 10.1002/hbm.23904 29193460 PMC6866356

[B32] Kuczynski A. M. Kirton A. Semrau J. A. Dukelow S. P. (2018b). Bilateral reaching deficits after unilateral perinatal ischemic stroke: a population-based case-control study. *J. Neuroeng. Rehabil.* 15:77. 10.1186/s12984-018-0420-9 30115093 PMC6097295

[B33] Lan Y. Yao J. Dewald J. P. A. (2017). The impact of shoulder abduction loading on volitional hand opening and grasping in chronic hemiparetic stroke. *Neurorehabil. Neural Repair* 31 521–529. 10.1177/1545968317697033 28506146 PMC5505320

[B34] Lawrence D. G. Kuypers H. G. (1968). The functional organization of the motor system in the monkey. I. The effects of bilateral pyramidal lesions. *Brain* 91 1–14. 10.1093/brain/91.1.1 4966862

[B35] Lynch J. K. Nelson K. B. (2001). Epidemiology of perinatal stroke. *Curr. Opin. Pediatr.* 13 499–505. 10.1097/00008480-200112000-00002 11753097

[B36] Mailleux L. Simon-Martinez C. Radwan A. Blommaert J. Gooijers J. Wenderoth N. (2020). White matter characteristics of motor, sensory and interhemispheric tracts underlying impaired upper limb function in children with unilateral cerebral palsy. *Brain Struct. Funct.* 225 1495–1509. 10.1007/s00429-020-02070-1 32318818

[B37] Martin J. H. (2005). The corticospinal system: from development to motor control. *Neuroscientist* 11 161–173. 10.1177/1073858404270843 15746384

[B38] McCall J. V. Ludovice M. C. Blaylock J. A. Kamper D. G. (2019). A platform for rehabilitation of finger individuation in children with hemiplegic cerebral palsy. *IEEE Int. Conf. Rehabil. Robot* 2019 343–348. 10.1109/ICORR.2019.8779537 31374653

[B39] McPherson J. G. Chen A. Ellis M. D. Yao J. Heckman C. J. Dewald J. P. A. (2018). Progressive recruitment of contralesional cortico-reticulospinal pathways drives motor impairment post stroke. *J. Physiol.* 596 1211–1225. 10.1113/JP274968 29457651 PMC5878212

[B40] McPherson L. M. Dewald J. P. A. (2019). Differences between flexion and extension synergy-driven coupling at the elbow, wrist, and fingers of individuals with chronic hemiparetic stroke. *Clin. Neurophysiol.* 130 454–468. 10.1016/j.clinph.2019.01.010 30771722 PMC7856836

[B41] Miller L. C. Dewald J. P. (2012). Involuntary paretic wrist/finger flexion forces and EMG increase with shoulder abduction load in individuals with chronic stroke. *Clin. Neurophysiol.* 123 1216–1225. 10.1016/j.clinph.2012.01.009 22364723 PMC3729226

[B42] Mockford M. Caulton J. M. (2010). The pathophysiological basis of weakness in children with cerebral palsy. *Pediatr. Phys. Ther.* 22 222–233. 10.1097/PEP.0b013e3181dbaf96 20473109

[B43] Netz J. Lammers T. Homberg V. (1997). Reorganization of motor output in the non-affected hemisphere after stroke. *Brain* 120(Pt 9) 1579–1586. 10.1093/brain/120.9.1579 9313641

[B44] Novak I. (2014). Evidence-based diagnosis, health care, and rehabilitation for children with cerebral palsy. *J. Child Neurol.* 29 1141–1156. 10.1177/0883073814535503 24958005

[B45] Palisano R. Rosenbaum P. Walter S. Russell D. Wood E. Galuppi B. (1997). Development and reliability of a system to classify gross motor function in children with cerebral palsy. *Dev. Med. Child Neurol.* 39 214–223. 10.1111/j.1469-8749.1997.tb07414.x 9183258

[B46] Penta M. Tesio L. Arnould C. Zancan A. Thonnard J. L. (2001). The ABILHAND questionnaire as a measure of manual ability in chronic stroke patients - Rasch-based validation and relationship to upper limb impairment. *Stroke* 32 1627–1634. 10.1161/01.Str.32.7.1627 11441211

[B47] Rosenbaum P. Paneth N. Leviton A. Goldstein M. Bax M. Damiano D. (2007). A report: the definition and classification of cerebral palsy April 2006. *Dev. Med. Child Neurol. Suppl.* 109 8–14. 17370477

[B48] Rosenbaum P. L. Palisano R. J. Bartlett D. J. Galuppi B. E. Russell D. J. (2008). Development of the gross motor function classification system for cerebral palsy. *Dev. Med. Child Neurol.* 50 249–253. 10.1111/j.1469-8749.2008.02045.x 18318732

[B49] Russo R. N. Skuza P. P. Sandelance M. Flett P. (2019). Upper limb impairments, process skills, and outcome in children with unilateral cerebral palsy. *Dev. Med. Child Neurol.* 61 1080–1086. 10.1111/dmcn.14185 30775778 PMC6850156

[B50] Sanger T. D. Chen D. Delgado M. R. Gaebler-Spira D. Hallett M. Mink J. W. (2006). Definition and classification of negative motor signs in childhood. *Pediatrics* 118 2159–2167. 10.1542/peds.2005-3016 17079590

[B51] Schwerin S. Dewald J. P. A. Haztl M. Jovanovich S. Nickeas M. MacKinnon C. (2008). Ipsilateral versus contralateral cortical motor projections to a shoulder adductor in chronic hemiparetic stroke: implications for the expression of arm synergies. *Exp. Brain Res.* 185 509–519. 10.1007/s00221-007-1169-8 17989973 PMC2831614

[B52] Schwerin S. C. Yao J. Dewald J. P. A. (2011). Using paired pulse TMS to facilitate contralateral and ipsilateral MEPs in upper extremity muscles of chronic hemiparetic stroke patients. *J. Neurosci. Methods* 195 151–160. 10.1016/j.jneumeth.2010.11.021 21134401 PMC3118562

[B53] Simon-Martinez C. Jaspers E. Mailleux L. Ortibus E. Klingels K. Wenderoth N. (2018). Corticospinal tract wiring and brain lesion characteristics in unilateral cerebral palsy: determinants of upper limb motor and sensory function. *Neural Plast.* 2018:2671613. 10.1155/2018/2671613 30344602 PMC6158964

[B54] Staudt M. (2010). Reorganization after pre- and perinatal brain lesions. *J. Anat.* 217 469–474. 10.1111/j.1469-7580.2010.01262.x 20649910 PMC2992421

[B55] Sukal T. M. Ellis M. D. Dewald J. P. (2007). Shoulder abduction-induced reductions in reaching work area following hemiparetic stroke: neuroscientific implications. *Exp. Brain Res.* 183 215–223. 10.1007/s00221-007-1029-6 17634933 PMC2827935

[B56] Sukal-Moulton T. Gaebler-Spira D. Krosschell K. J. (2018). The validity and reliability of the test of arm selective control for children with cerebral palsy: a prospective cross-sectional study. *Dev. Med. Child Neurol.* 60 374–381. 10.1111/dmcn.13671 29383702 PMC5867232

[B57] Sukal-Moulton T. Krosschell K. J. Gaebler-Spira D. J. Dewald J. P. (2014a). Motor impairment factors related to brain injury timing in early hemiparesis. Part I: expression of upper-extremity weakness. *Neurorehabil. Neural Repair* 28 13–23. 10.1177/1545968313500564 24009182 PMC3974904

[B58] Sukal-Moulton T. Krosschell K. J. Gaebler-Spira D. J. Dewald J. P. (2014b). Motor impairments related to brain injury timing in early hemiparesis. Part II: abnormal upper extremity joint torque synergies. *Neurorehabil. Neural Repair* 28 24–35. 10.1177/1545968313497829 23911972 PMC3974905

[B59] Yeo S. S. Chang M. C. Kwon Y. H. Jung Y. J. Jang S. H. (2012). Corticoreticular pathway in the human brain: diffusion tensor tractography study. *Neurosci. Lett.* 508 9–12. 10.1016/j.neulet.2011.11.030 22197953

